# Structural studies on molecular mechanisms of Nelfinavir resistance caused by non-active site mutation V77I in HIV-1 protease

**DOI:** 10.1186/1471-2105-16-S19-S10

**Published:** 2015-12-16

**Authors:** Ankita Gupta, Salma Jamal, Sukriti Goyal, Ritu Jain, Divya Wahi, Abhinav Grover

**Affiliations:** 1 Department of Biotechnology, Delhi Technological University, New Delhi, India; 2 Department of Bioscience and Biotechnology, Banasthali University, Tonk, Rajasthan, India; 3 School of Biotechnology, Jawaharlal Nehru University, New Delhi, India

**Keywords:** HIV, Nelfinavir, mutation, mechanism, resistance, docking, molecular dynamics simulations

## Abstract

**Background:**

The human immunodeficiency virus (HIV-1) is a retrovirus causing acquired immunodeficiency syndrome (AIDS), which has become a serious problem across the world and has no cure reported to date. Human immunodeficiency virus (HIV-1) protease is an attractive target for antiviral treatment and a number of therapeutically useful inhibitors have been designed against it. The emergence of drug resistant mutants of HIV-1 poses a serious problem for conventional therapies that have been used so far. Until now, thirteen protease inhibitors (PIs), major mutation sites and many secondary mutations have been listed in the HIV Drug Resistance Database. In this study, we have studied the effect of the V77I mutation in HIV-PR along with the co-occurring mutations L33F and K20T through multi-nanosecond molecular dynamics simulations. V77I is known to cause Nelfinavir (NFV) resistance in the subtype B population of HIV-1 protease. We have for the first time reported the effect of this clinically relevant mutation on the binding of Nelfinavir and the conformational flexibility of the protease.

**Results:**

Two HIV-PR mutants have been considered in this study - the Double Mutant Protease (DBM) V77I-L33F and Triple Mutant Protease (TPM) V77I-K20T-L33F. The molecular dynamics simulation studies were carried out and the RMSD trajectories of the unliganded wild type and mutated protease were found to be stable. The binding affinity of NFV with wild type HIV-PR was very high with a Glide XP docking score of -9.3 Kcal/mol. NFV showed decreased affinity towards DBM with a docking score of -8.0 Kcal/mol, whereas its affinity increased towards TPM (Glide XP score: -10.3). Prime/MM-GBSA binding free energy of the wild type, DBM and TPM HIV-PR docked structures were calculated as -38.9, -11.1 and -42.6 Kcal/mol respectively. The binding site cavity volumes of wild type, DBM and TPM protease were 1186.1, 1375.5 and 1042.5 Å^3 ^respectively.

**Conclusion:**

In this study, we have studied the structural roles of the two HIV-PR mutations by conducting molecular dynamics simulation studies of the wild type and mutant HIV-1 PRs. The present study proposes that DBM protease showed greater flexibility and the flap separation was greater with respect to the wild type protease. The cavity size of the MD-stabilized DBM was also found to be increased, which may be responsible for the decreased interaction of Nelfinavir with the cavity residues, thus explaining the decreased binding affinity. On the other hand, the binding affinity of NFV for TPM was found to be enhanced, accounted for by the decrease in cavity size of the mutant which facilitated strong interactions with the flap residues. The flap separation of TPM was less than the wild type protease and the decreased cavity size may be responsible for its lower resistance, and hence, may be the reason for its lower clinical relevance.

## Background

Human immunodeficiency virus type 1 (HIV-1), a member of the retrovirus family, causes acquired immunodeficiency syndrome (AIDS) which progressively destroys the body's natural immune system leading to deadly infections and cancers [1]. According to the World Health Organization (WHO), HIV has affected 35 million people so far and continues to be a major public health burden globally, with 69% of the population affected alone in sub-Saharan Africa [2,3]. The current therapeutic options available include highly active antiretroviral therapy (HAART) combination, a set of antiretroviral drugs which inhibit the replication of virus in the body and reduce the burden of the disease, but drugs or vaccines which can eradicate the viruses from the human body still remain a question to be addressed [4]. Additionally, the existing antiretroviral drugs are very expensive and are associated with the risk of non-AIDS disorders that include cardiovascular, liver, kidney and neurological disease [5]. Owing to the huge global burden of the disease, the lack of effective drugs and vaccines which can kill the pathogen, the high costs and the serious side effects related to the existing drugs and the accumulating drug resistance has made the search for anti-HIV drugs a foremost research priority.

**Figure 1:**
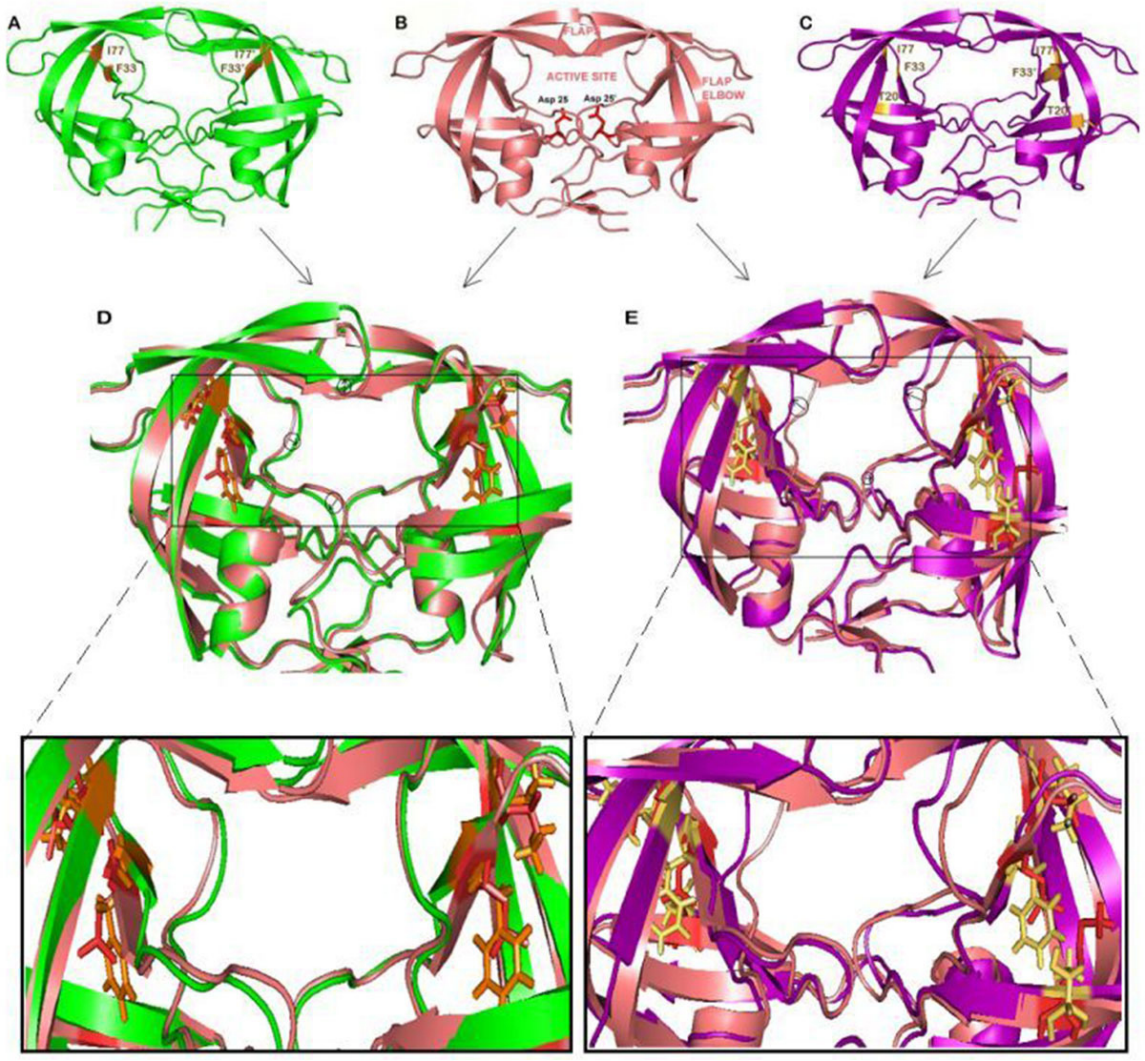
**(A) Structure of double mutated protease: DBM**. Mutated residues V77I and L33F are shown in yellow. (B) Crystal structure of HIV-1 PR. (C) Structure of triple mutated protease: TPM. Mutated residues V77I, L33F and K20T are shown in yellow. (D) DBM superimposed on wild type HIV-1 PR. (E) TPM superimposed on wild type HIV-1 PR. Difference between wild and mutants is highlighted using arrows and circles. The superimposed structure are further magnified to view the side chains.

HIV-1 proliferates with the support of its own homodimeric aspartic protease, an enzyme essential for viral replication and assembly, also referred to as HIV-1 protease (HIV-1 PR) [2]. HIV1-PR is a homodimer of a 99 amino acid long sequence that forms C2 symmetry in the absence of the natural substrate or ligand [3]. The dimer interface forms the active site of the enzyme, which have two catalytic aspartic acid residues. The function of protease is assisted by the characteristic flap movement which provides restricted access to the active site. The flaps are flexible, antiparallel, glycine-rich β-sheets made of residues 45-55 from both the chains of the homodimer (Figure 1B) [4-6]. From X-ray crystallographic studies it is known that there are consistent structural differences between the bound and free-state of the protein. The flaps adopt a semi-open conformation in the unbound state, whereas they are pulled into the active site to form a closed structure in the bound state [7,8]. The recognition of HIV-1 PR as a major target for antiviral therapy has led to determination of its large number of structures with slight sequence variations and different ligands. HIV-1 PR cleaves the nonfunctional polyproteins that include gag and pol proteins, reverse transcriptase, integrase, and protease itself, and is responsible for creating the mature and functional components of the protein [9].

Functional inhibition of HIV-1 PR leads to incomplete viral replication and therefore makes it an attractive target for anti-HIV drugs [3]. To date, seven protease inhibitors (PIs) have been approved by the U.S. Food and Drug Administration [10]. However, emergence of several resistant viral species due to genetic mutations in active and non-active sites of HIV-1 PR has made the current therapies inefficient and inappropriate for use. The active site mutations also referred to as primary mutations cause direct resistance, while the secondary mutations often accompany primary mutations (accessory mutations) or they show synergistic resistance in the presence of other secondary mutations [11]. These mutations are a consequence of the selective pressure rising due to antiviral agents. Another driving force of the resistance causing mutations is the recently reported immunological pressure and the mutations are described as 'Escape mutations' [12].

V77I is one of the important non active site secondary mutations in HIV-PR, causing resistance against Nelfinavir (NFV). It is a highly polymorphic mutation present near the cheek sheet of protease, with a marked presence in subtype B virus. This minor mutation is accompanied by other primary and secondary mutations [13]. L33F is a major mutation present in the active site of the protease [14], which results in reduced susceptibility towards NFV in the presence of other mutations. It is a non-polymorphic mutation which provides resistance against all PIs except Indinavir and Saquinavir. L33I is a less commonly occurring mutation with similar effects to L33F, while L33V mutation has not been related to any kind of drug resistance in PI therapy. L33F co-occurs with V77I in large number of HIV-1 subtype B infected patient samples, as reported in Stanford's HIV Drug Resistance Database [13]. The mutation at the 20^th ^residue is another non-polymorphic site present in the cheek turn and is involved in rendering resistance against all PIs except Saquinavir and Tipranavir. K20T is the most prominent mutation occurring at the 20^th ^residue [15] and has been found to co-occur with V77I in subtype B population. It is interesting to note that the 77^th^, 33^rd ^and 20^th ^amino acids form a set of residues interacting with the 36^th ^residue of the protease, which itself is present on non-active site and its mutation causes resistance to NFV in non-subtype B viruses [16]. L23I, D30N, E35G, M46I/L/V, G48V, I54L, G73S/T/C/A, T74S, V82A/F/S/T, I84V, N88D/S and L90M are other mutations correlated to NFV resistance.

In the present study, we have scrutinized the behavior of the minor mutation V77I along with co-occurring mutations L33F and K20T and have considered the two types of mutants, a double mutant, V77I-L33F (DBM) and a triple mutant, V77I-L33F-K20T (TPM); according to their actual prevalence [13] (Table 1). The other mutations which were co-occurring with DBM and TPM included major mutations like- M46IL, I54MV, I84V, L90M, N88S, V32I, I47V, V82AS, D30N, G48V; and accompanied by minor mutations like- L10I, I13V, L63P, A71V, L89M, I93L, E35DN, I15V, D60E, L24I etc. We have studied the structural roles of these mutations at molecular levels with the aid of molecular dynamics simulations of the wild type and mutant HIV-1 PRs in their unbound NFV-bound docked complexes. Molecular dynamics simulation has become one of the most important tools in the theoretical study of biological molecules. This computational approach calculates the time dependent behavior of a molecular system and can provides even minute details related to fluctuations and conformational changes that may occur in the protein structure. This method is now being used routinely to investigate the structure, dynamics and thermodynamics of biological molecules and their complexes [17,18]. The protease-ligand (NFV) interaction energies were calculated for wild type and mutant proteases using MM/GBSA approach. Similar kind of studies on other biological systems reporting the underlying molecular mechanisms of drug resistance have been successfully carried out and reported before and have provided valuable insights into the comparative mode of interactions of drugs with mutants and wild type proteins [19-25].

**Table 1:** Number of subtype-b clinical isolates reported in HIV drug resistance database

MUTANT NAME	MUTATION SET	ISOLATES
**DBM**	V77I-L33F	407
**TPM**	V77I-L33F-K20T	16

## Materials and methods

### Preparation of wild type and mutated HIV-1 protease structures

The crystal structure of protease-NFV complex (PDB: 1OHR) [26] was downloaded from the Protein Data Bank [27] and was pre-processed using ViewerLite, a visualizing tool from Accelrys (Accelrys, Inc., San Diego, CA, USA). The ligand NFV and the water molecules were removed from the structure and the protein was further prepared and optimized using Schrödinger's protein preparation wizard [28,29]. The residues V77 and L33 were mutated to isoleucine and phenyalanine to obtain double mutant DBM (Figure 1A) through the protein preparation wizard. Similarly triple mutant TPM (Figure 1C) was obtained by mutating residues V77, L33 and K20 to isoleucine, phenylalanine and threonine respectively. The preparation of structures involved addition of hydrogen bonds, creation of disulfide bonds, removal of bad contacts, capping of protein terminals, optimization of bond lengths, conversion of selanomethionine to methionine and cleaning the geometry of overlapping residues. Side chain prediction and refinement of selected residues was carried out using PRIME module of Schrodinger (Prime, version 2.1, Schrödinger, LLC, New York, NY, 2009). The study of flap movements is crucial to understand and compare the molecular dynamics of wild type and mutants HIV-1 PR. In the ligand-bound crystal structure, the flap residues were involved in interactions with the inhibitor or natural substrate [30,31] while the flaps of unliganded protease were found to be highly flexible [32]. To analyze the flap motions of our mutants with respect to the wild type protease, we selected the unliganded structure [33] of HIV-1 protease [PDB: 1HVP]. The double (DBM_M) and triple (TPM_M) mutants were prepared similarly to the procedure described above.

The drug Nelfinavir (NFV) (CID 64143), was processed before docking using LigPrep's ligand preparation protocol (Ligprep v2.5; Schrödinger, Inc.: Portland, OR, 2011). The three-dimensional coordinates (tautomeric, stereochemical, ionizing variants) were generated along with their energy minimization and flexible filtering.

### Molecular dynamics simulations

MD simulations of the docked and unliganded complexes (both wild type and mutant) were accomplished using Desmond Molecular Dynamics system, with Optimized Potentials for Liquid Simulations (OPLS) all-atom force field 2005 [34,35] as described in some of our previous studies [36-39]. The prepared protein molecules were solvated in the presence of explicit solvent on a fully hydrated model with TIP4P water model in a triclinic periodic boundary box (distance between box wall and protein complex was kept at 10 Å to avoid the direct interaction with its own periodic image) to generate required systems for MD simulations. The energy of prepared systems for MD simulations was minimized to 5000 steps maximum using the steepest descent method until a gradient threshold (25 kcal/mol/Å) was reached, followed by L-BFGS (Low-memory Broyden-Fletcher- Goldfarb Shanno quasi-Newtonian minimizer) until a convergence threshold of 1 kcal/mol/Å was met. The default parameters in Desmond were applied for systems equilibration. The so equilibrated systems were then used for simulations at 300 K temperature and a constant pressure of 1atm, with a time step of 2fs. The long range electrostatic interactions were handled using Smooth Particle Mesh Ewald Method. Cutoff method was selected to define the short range electrostatic interactions. A cutoff of 9 Å radius (default), was used.

The prepared conformations of NFV were docked to the stabilized mutants DBM and TPM using Glide docking software [40,41]. We performed semi-flexible docking where the ligand was made flexible keeping the receptor macromolecule rigid. The flexibility of ligand molecule gives it the freedom to search from six degrees of rotational and translational freedom and an arbitrary number of torsional degrees of freedom. A random perturbation to each was applied at each time step, and the interaction energy was evaluated for the new location and conformation.

A scoring grid was prepared on the active site of the homodimer i.e. at the interface of both the subunits, using receptor grid generation platform of Schrödinger [40,41]. Keeping all the parameters default, a grid of size 20 × 20 × 20 Å with an inner box size of 10 × 10 × 10 Å was generated.

### Calculation of binding energies using MM/GBSA

The binding free energy was calculated according to the Generalized Born Model and Solvent Accessibility method, using Prime MM/GBSA [42] (Prime version 2.1, 2009). NFV-docked protease structures were used for calculation of free energy of the wild type and mutant structures. The binding free energy ΔG_binding _was calculated using the following equation:

$$\Delta {\text{G}}_{\text{binding}}={\text{E}}_{\text{R}:\text{L}}-\left({\text{E}}_{\text{R}}+{\text{E}}_{\text{L}}\right)+\Delta {\text{G}}_{\text{SA}}+\Delta {\text{G}}_{\text{SOLV}};$$

Where E_R _+ E_L _is the sum of energies of unbound ligand and receptor, and E_R:L _is the energy of the docked complex. ΔG_SA _is the difference of surface area energy of the protein-ligand complex and the sum of surface area energies of protein and ligand individually. ΔG_SOLV _is the difference in the GBSA solvation energy of the complex and summation of individual salvation energies of protein and ligand. Energies of the complex were calculated using the OPLS-All Atom force field [35] and GB/SA continuum solvent model.

### Hydrogen bond and hydrophobic interaction analysis

The hydrophobic interactions and H-bonds of the docked complexes were analysed using the Ligplot program [43]. The parameters defining the H-bonds between ligand and the protein complexes were as follows: acceptor-donor atoms distances less than 3.3 Å, hydrogen acceptor atom distances less than 2.7 Å and an acceptor-donor angle of 90° or more. Ligand-bound protease structures obtained from Glide and the MD-stabilized representative structures from Desmond were selected for carrying out interaction studies. A representative structure was prepared by averaging the coordinates of various frames extracted from the most stable region of the trajectory, which persisted until the end of the simulation run.

### SYFPEITHI epitope prediction analysis

The human immune system proteins human leukocyte antigens (HLAs) bind to the intracellular epitopes arising from digestion of viral proteins. HLAs are responsible for presenting these epitopes on the cell surface and hence triggering an immune response against the virus. Escape mutations hinder strong binding of HLA to the epitopes and thereby assist in bypassing the immune response. Epitope prediction was done using Syfpeithi Database of MHC ligands and peptide motifs [44]. The algorithm relies on the scoring of binding motifs. From the first amino acid of the protein, its sequence is divided into octamers, nonamers and decamers. The score of each oligomer is then calculated according to the summation of scores of individual amino acids. The amino acids are scored based on their observed frequencies. Most frequently occurring residues in the anchor positions are given a value 10, followed by a value of 8 given to the residues occurring in significant number of ligands. Likewise, residues regarded unfavorable for binding have a coefficient of -1 to -3. We have used MHC class HLA-A3 for our analysis.

### Calculation of volume and surface area of HIV-1 PR cavity

We used CASTp server [45] to estimate the cavity volume and surface area of wild type and mutant proteases. CASTp works on the principles of Alpha Shape Theory [46] for detection and measurement of pockets in a protein which are inaccessible to the solvent outside. A probe of radius 1.4 Å was used for cavity measurement.

## Results and discussion

### Molecular dynamics simulations and energy stabilization of unliganded wild type, DBM and TPM protease

**Figure 2:**
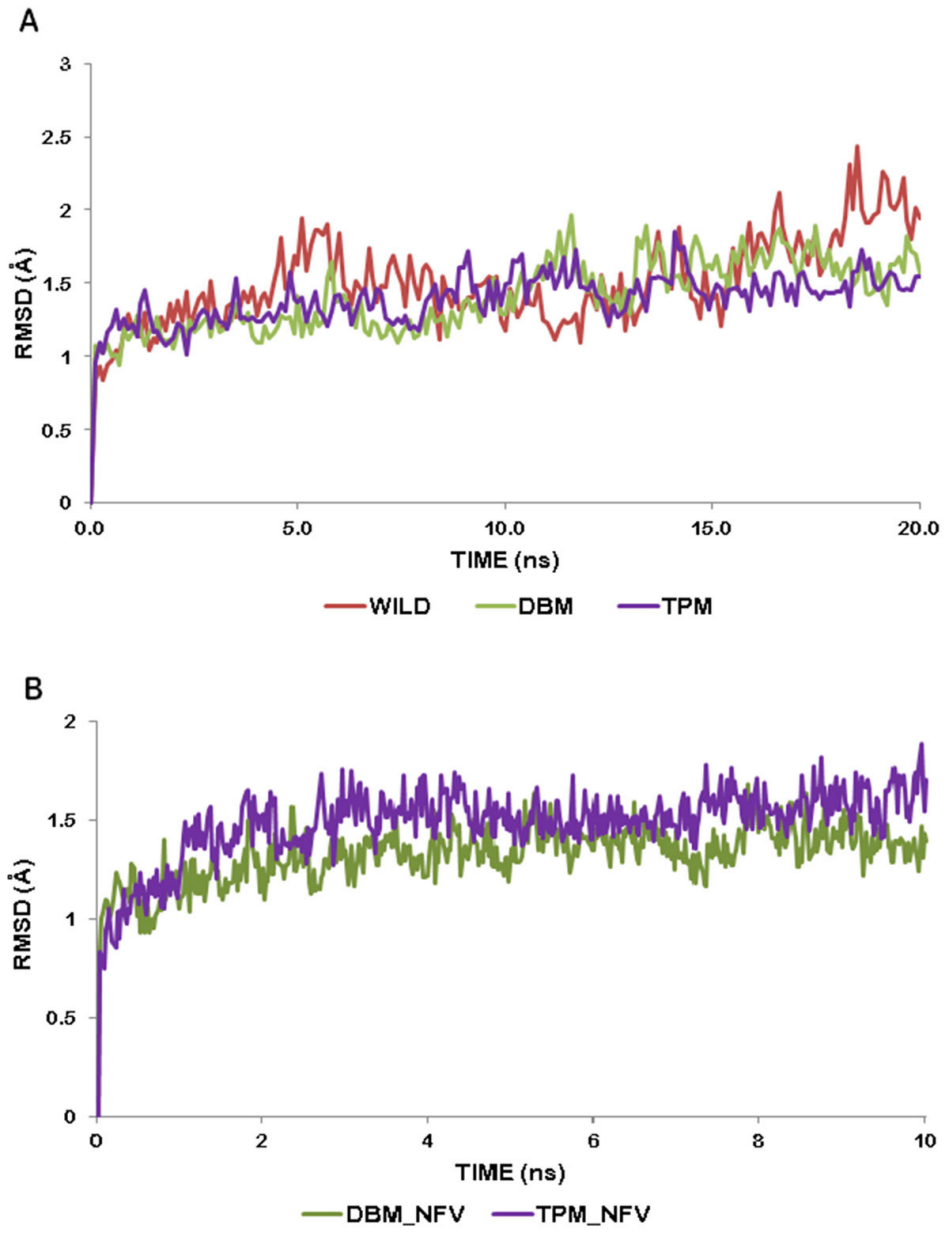
**(A) RMSD trajectory of Wild protease, DBM and TPM during MD simulations**. Trajectory for Wild protease (red line), DBM (green line) and TPM (purple line). (B) RMSD trajectory of NFV docked DBM and TPM during MD simulations. Trajectory for DBM (green line) and TPM (purple line).

**Figure 3:**
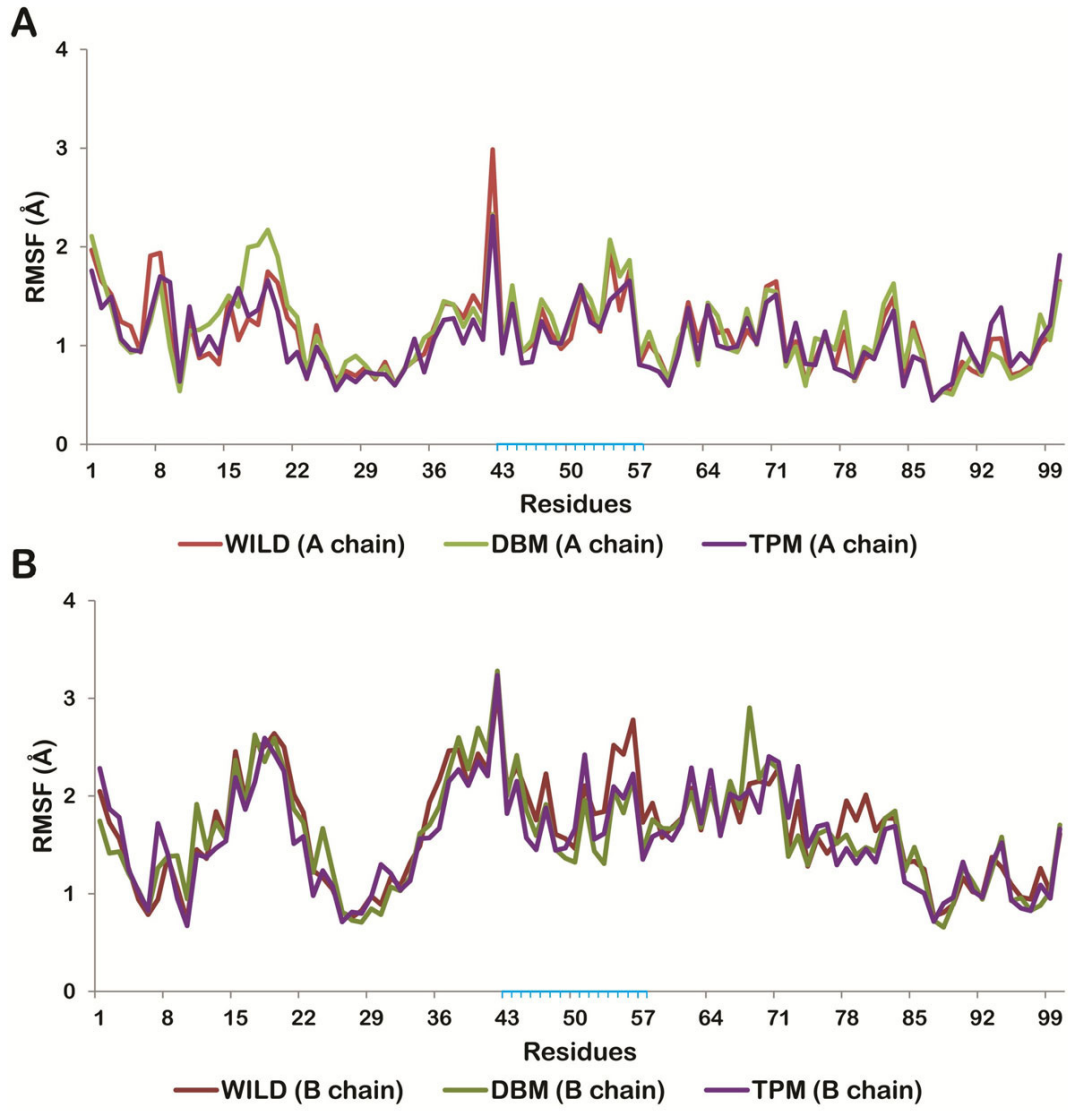
**Residue wise RMS fluctuations of Wild protease (red line), DBM (green line) and TPM (purple line)**. (A) chain A. (chain B). Residues present in flap region are highlighted in blue.

To study the structural changes in closed conformation of HIV-1 PR due to mutation we have considered the NFV-docked crystal structure of HIV-1 PR. NFV was removed before mutating the residues and then MD simulations of unliganded wild type, double and triple mutants were carried out for 20 ns. The Root Mean Square Deviation (RMSD) of DBM and TPM were found to be more stable with respect to that of wild protease, with standard deviations of 0.28, 0.23 and 0.16 for wild type, DBM and TPM respectively (Figure 2). To observe and compare the movement of residues, we plotted the Root Mean Square Fluctuation (RMSF) plot for both the subunits of the wild type and mutant proteases (Figure 3). RMSF is a measure of average atomic mobility of the backbone atoms during the MD simulations. It was observed that the residues of DBM deviated more from the wild type than that of TPM protease, especially in chain A (Figure 3A). It was observed that the flap residues (33-62) of the wild type protease were more flexible in comparison to the mutants DBM and TPM, indicating that there were relatively strong interactions between the flaps of the mutants which made them more stable in close conformation than the wild type protease.

The representative structures from the MD simulation trajectories of DBM and TPM were then selected for studying their interactions with NFV. The mutants were docked with all the stable conformations of NFV using Glide and were compared with the NFV-docked crystal structure of wild type HIV-1 PR.

### Docking of NFV with wild type protease

**Figure 4:**
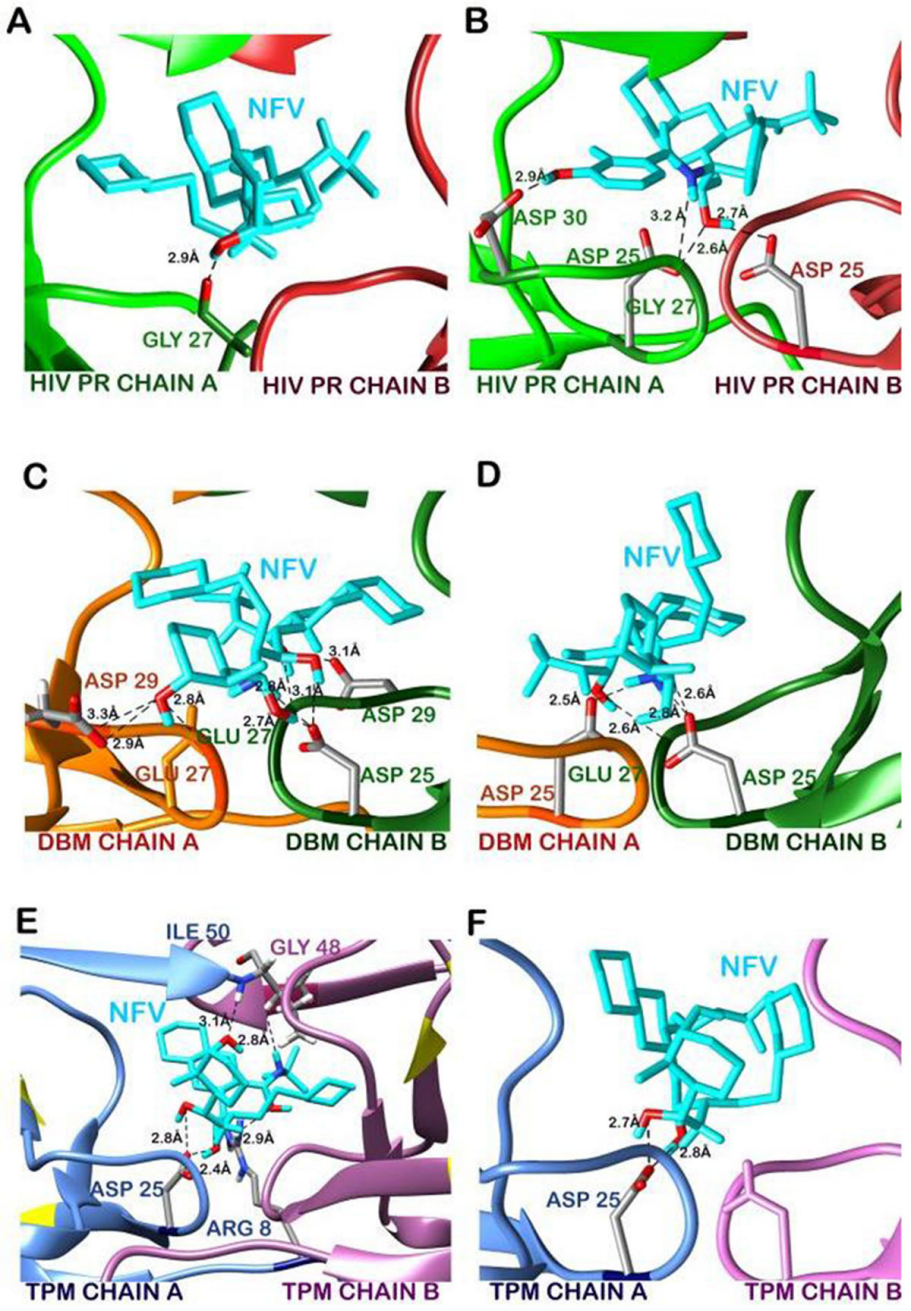
**Changes in the hydrogen bonds of NFV with protease before and after simulation**. A. hydrogen bonds with wild protease in Glide docked structure. B. in PDB structure 1OHR. C. hydrogen bonds with DBM before simulation and D. after simulation. E. hydrogen bonds with TPM before simulation and F. after simulation. (Oxygen-Red, Nitrogen-Blue, Carbon-Grey)

**Figure 5:**
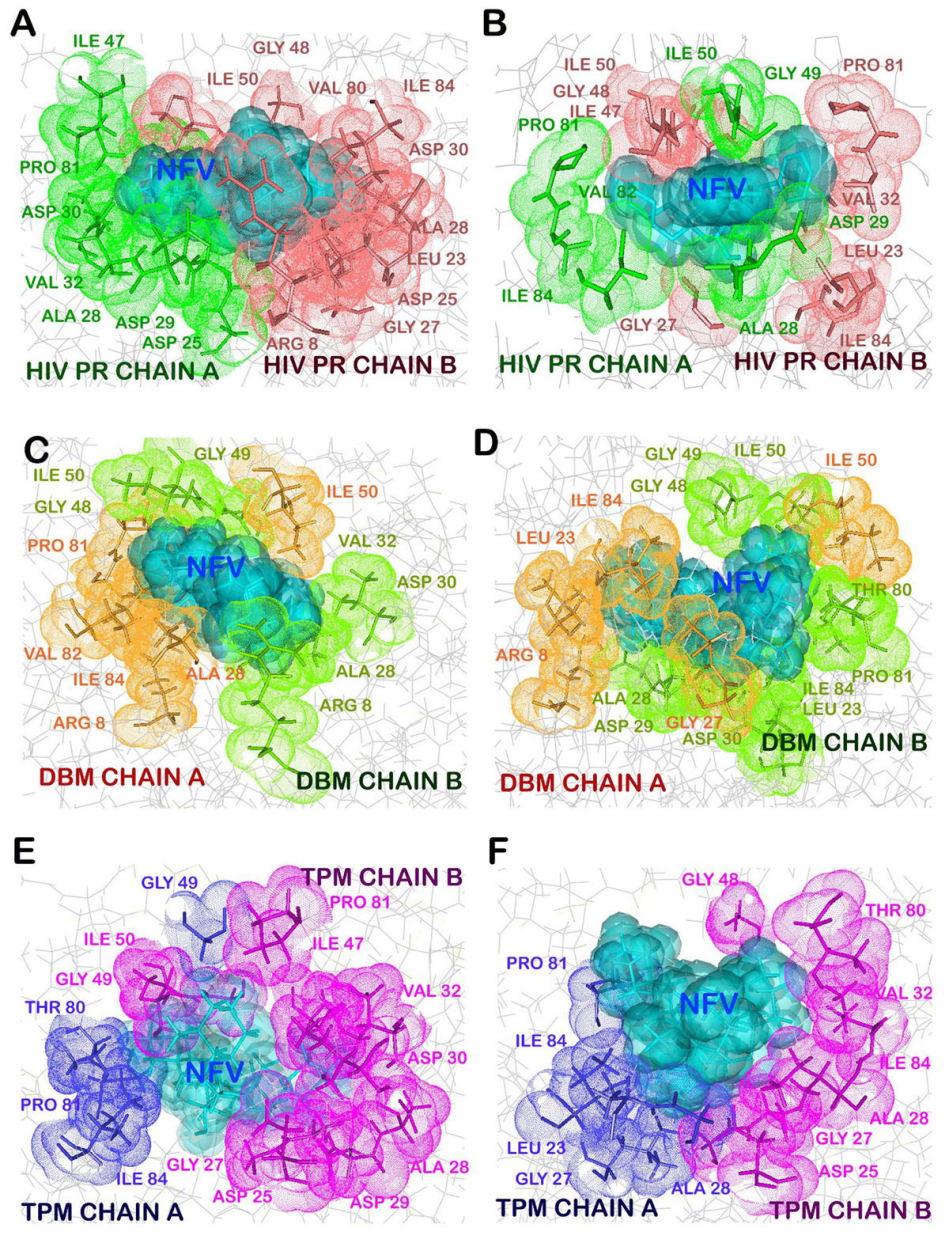
**Changes in the hydrophobic interactions of NFV with protease before and after simulation**. A. hydrophobic interactions with wild protease in Glide docked structure. B. in PDB structure 1OHR. C. hydrophobic interactions with DBM before simulation and D. after simulation. E. hydrophobic interactions with TPM before simulation and F. after simulation.

The wild type protease showed a high affinity with NFV, with an XP docking score of -9.32 Kcal/mol (Table 2). This strong interaction was mediated by a number of hydrophobic interactions from both the chains of wild type proteases and a hydrogen bond between Gly27 of A chain of protease with oxygen atom of NFV (Figure 4A, 5A). Prime/MM-GBSA free binding energy of the wild type docked structure was calculated to be -38.98kcal/mol. We compared these interactions between NFV and wild type protease with the reference crystal structure of the protease. In this case, stronger interactions were observed which were accounted for by a large number of hydrophobic interactions (Figure 5B) from both the chains of protease in addition to 4 hydrogen bonds. The catalytic site residues Asp25 (A) and Asp25 (B) were found forming hydrogen bonds with NFV through their delta oxygen atoms. The delta oxygen atom of Asp 30(A) was involved in forming a 2.90Å long hydrogen bond with NFV, while the nitrogen atom of NFV formed hydrogen bond with Gly27 (A) (Figure 4B). Table 3 shows residues involved in hydrophobic interactions in 1OHR and Glide docked structure.

**Table 2:** Docking score

Docking Score	Wild	DBM	TPM
**Glide XP**	-9.32	-8.04	-10.31
**MM/GBSA(Kcal/mol)**	-38.98	-11.08	-42.66

**Table 3:** Hydrophobic interactions

WILD PROTEASE	
**GLIDE DOCKED**	**1OHR**

Arg 8(B), Leu 23(B), Gly 27(B), Asp 25 (A)(B), Ala 28 (A)(B), Asp 29(A)(B), Asp 30 (A)(B), Val 32(A), Ile 47(A), Gly 48(B), Ile 50 (B), Val 82(B), Ile 84(B)	Leu 23(B), Gly 27(B), Ala 28 (A), Asp 29(A), Val 32(B), Ile 47(B), Gly 48(B), Gly 49(B), Ile 50 (A)(B), Val 82(A)(B), Pro 81(A)(B), Ile 84(A)(B)

**DBM**	

**GLIDE DOCKED**	**After MD Simulation**

Arg 8(A)(B), Ala 28(A)(B), Asp 30(B), Val 32(B), Ile 47(A), Gly 48(B), Gly 29(B), Ile 50 (A)(B), Pro 81(A), Val 82(A), Ile 84(A)	Arg 8(A), Leu 23(A)(B), Gly 27(A), Ala 28 (A)(B), Asp 29(B), Asp 30(B), Gly 48(B), Ile 49(B), Ile 50(A), Ile 54(B), Thr 80(B), Phe 81(B), Ile 84(A)(B)

**TPM**	

**GLIDE DOCKED**	**After MD Simulation**

Gly 27(B), Asp 25(B), Ala 28 (B), Asp 29(B), Asp 30(B), Val 32(B), Ile 47(B), Gly 49(A)(B), Ile 50 (B), Thr 80(A), Val 81(A)(B), Ile 84(A)(B)	Leu 23(A), Gly 27(A)(B), Asp 25(B), Ala 28 (A)(B), Val 32(B), Gly 48(B), Val 82(B), Pro 81(A), Ile 84(A)

### Docking of NFV with DBM and energy stabilization of NFV-DBM docked complex

**Figure 6:**
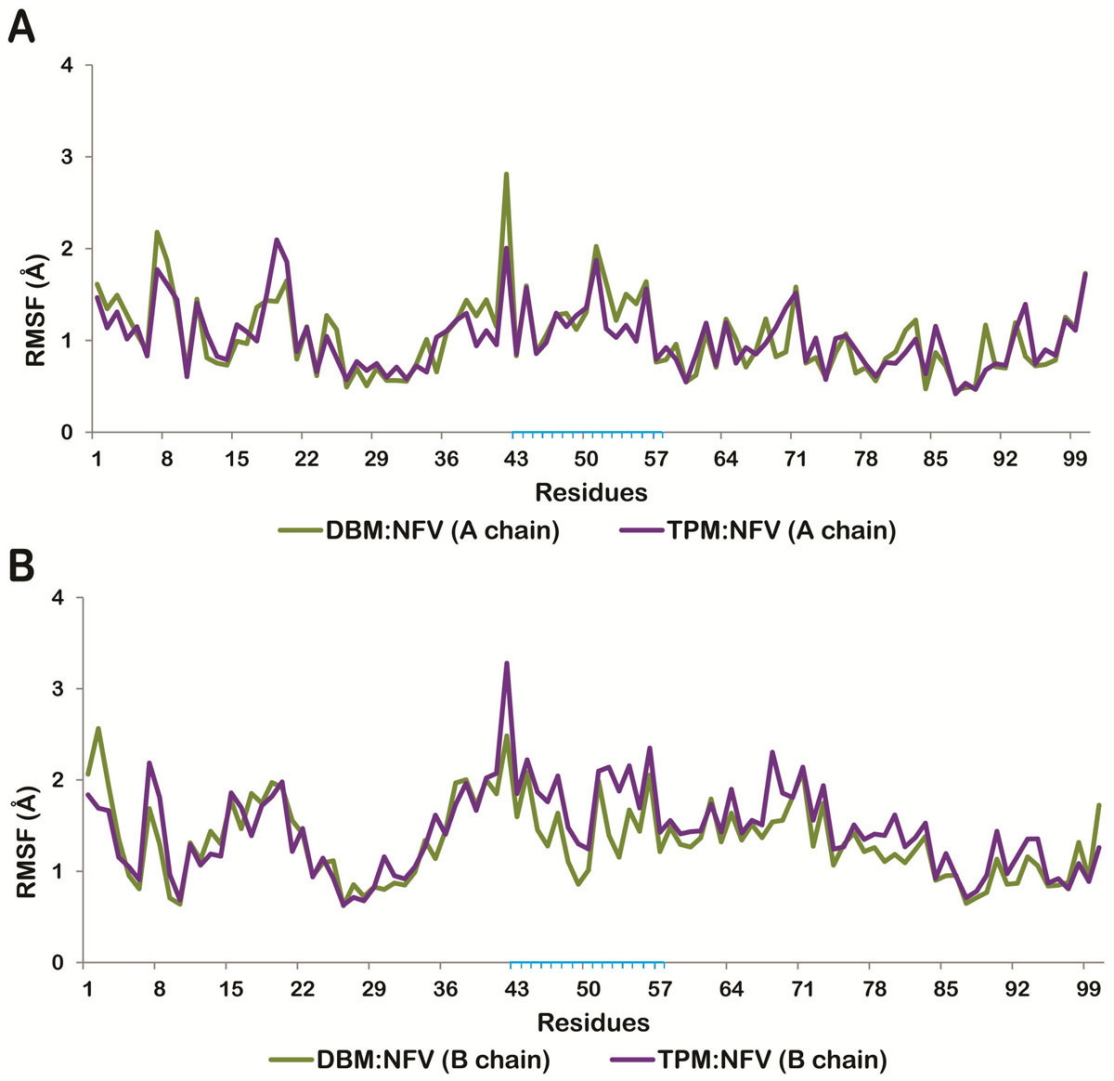
**Residue wise RMS fluctuations of NFV docked-DBM (green line) and TPM (purple line)**. (A) chain A (B) chain B. Residues present in flap region are highlighted in blue.

Similar strategy was applied to study the binding interactions of the two mutants DBM and TPM. The binding affinity of NFV to DBM was found to be lowered with a docking score of -8.04 Kcal/mol. The Prime/MM-GBSA binding free energy of NFV-docked DBM was also found to be significantly decreased by 27.90 Kcal/mol to -11.08 Kcal/mol (Table 2). Binding of NFV was mediated through several hydrophobic interactions from both the chains of DBM and also involved six hydrogen bond interactions between protease and NFV (Figure 4C, 5C). These H-bonds were formed by residues Gly27 (A), Asp29 (A), Gly27 (B), Asp25 (B) and Asp29 (B) (Figure 4C). The NFV-DBM docked structure was stabilized using molecular dynamics simulations of 10ns. The structure was found to be stable throughout the simulations with a low RMSD of 0.132 (Figure 2B). A decrease in the flexibility of flap and active site residues was observed in NFV-bound DBM as compared to the undocked DBM (Figure 6). The binding interactions were reduced to only four distant hydrogen bonds and few weak hydrophobic interactions. It was observed that the alignment of NFV also got perturbed, reducing its interactions with the cavity residues. It was found that the hydrogen bonds were formed by active site residues of B chain only which were Asp25(B) and Gly27(B) (Figure 4D). The residues mediating hydrophobic interactions before and after MD simulations are mentioned in Table 3.

### Docking of NFV with TPM and energy stabilization of NFV-TPM docked complex

The results of docking and simulations studies of triple mutant TPM were notably different from other resistant proteases and therefore justified its lower clinical presence with respect to DBM. The docking scores of NFV with stable mutant TPM with NFV was found to be -10.31 Kcal/mol. The Prime/MM-GBSA free binding energy was stabilized by 3.68 Kcal/mol to -42 Kcal/mol with respect to the wild type protease. This binding involved five hydrogen bonds with residues Arg 8(A), Asp 25(A), Ile 50(A) and Gly 48(B) along with a number of hydrophobic interactions (Figure 4E, 5E). However, the number of interactions got significantly reduced after MD simulations. The NFV-TPM complex was found to be stable throughout the simulations trajectory with RMSD of only 0.183. The RMSF values of individual residues were also found lowered with respect to the undocked TPM. NFV was observed forming only two hydrogen bonds which were made with Asp 25(A). From this it becomes evident that the otherwise resistant K20T mutation has the potential to reduce the resistance of TPM in comparison to DBM (Figure 4F, 5F).

### Possible intra-molecular interactions by V77I, L33F, K20T and the neighboring residues, and their effect on cavity size

To further investigate the role of these clinically significant mutations on the structure of the protease and to understand the reasons behind the marked variation in interactions and docking scores, the cavity size and volume of the mutants and wild protease were studied. The cavity volume and area were calculated at 0 ns, 5 ns, 10 ns, 15 ns and 20 ns of MD simulations (Table 4). The pocket volume and surface area of wild type protease (1OHR) were found to be 1186.1 Å^3 ^and 705.9 Å^2 ^respectively. We analyzed the number of probable hydrogen bonds formed by the mutated and their neighboring residues. Lys20 of both the wild type protease chains formed two hydrogen bonds with their corresponding Ile13 residues. Leu33 formed hydrogen bonds with residues Leu76 and Gly78 of both the chains. Val77 formed two hydrogen bonds with Arg57 in both the chains.

**Table 4:** Binding cavity size and area

WILD						
**CAVITY**	**0 ns**	**5 ns**	**10 ns**	**15 ns**	**20 ns**	**1OHR**

**VOLUME (Å^3^)**	1186.1	976.3	990.3	113.3	1097.3	1186.1
**AREA (Å^2^)**	705.9	612.3	734.6	739.645	645.3	705.9

**DBM**						

**CAVITY**	**0 ns**	**5 ns**	**10 ns**	**15 ns**	**20 ns**	**Representative**

**VOLUME (Å^3^)**	1156.0	790.6	1271.5	1403.2	1024.1	1375.5
**AREA (Å^2^)**	686.2	505.4	697.7	771.6	757.0	732.1

**TPM**						

**CAVITY**	**0 ns**	**5 ns**	**10 ns**	**15 ns**	**20 ns**	**Representative**

**VOLUME (Å^3^)**	1156.0	1257.8	938.3	820.2	801.2	1042.5
**AREA (Å^2^)**	686.2	753.9	554.8	572.9	498.5	634.3

V77I mutation in combination with L33F (DBM) displayed increase in the size of binding cavity (representative structure) with volume and area as 1375.5 Å^3 ^and 732.10 Å^2 ^respectively. This increase in cavity size is the probable reason behind decreased binding affinity of NFV to DBM (due to decrease in contact surface area of ligand and active site residues). L33F mutation caused positional change of neighboring residue Glu34, which resulted in formation of an extra hydrogen bond between Glu34 and Lys20 in DBM.

The binding pocket volume and area of the triple mutant representative structure got reduced with respect to the wild type protease, thereby increasing the contact surface area between ligand and active site residues. The pocket volume and surface area of TPM was found to be 1042.5 Å^3 ^and 634.3 Å^2 ^respectively. Thr20 formed an additional hydrogen bond with Gly21 in B chain, whereas hydrogen bonds between 33rd residue and Gly34 in B chain, and Leu76 in A chain were lost as a result of mutations.

### Comparison of flap movements of double and triple mutant with the wild type protease

**Figure 7:**
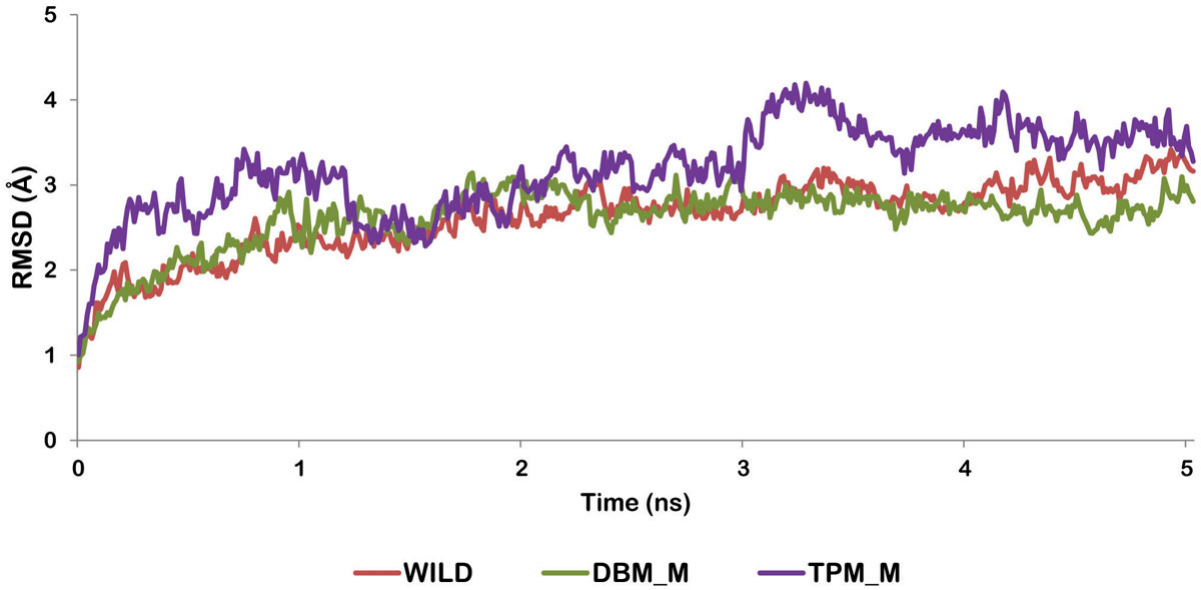
**RMSD trajectory of Modeled Wild protease, DBM_M and TPM_M during MD simulations**. Trajectory for Wild protease (red line), DBM_M (green line) and TPM_M (purple line).

**Figure 8:**
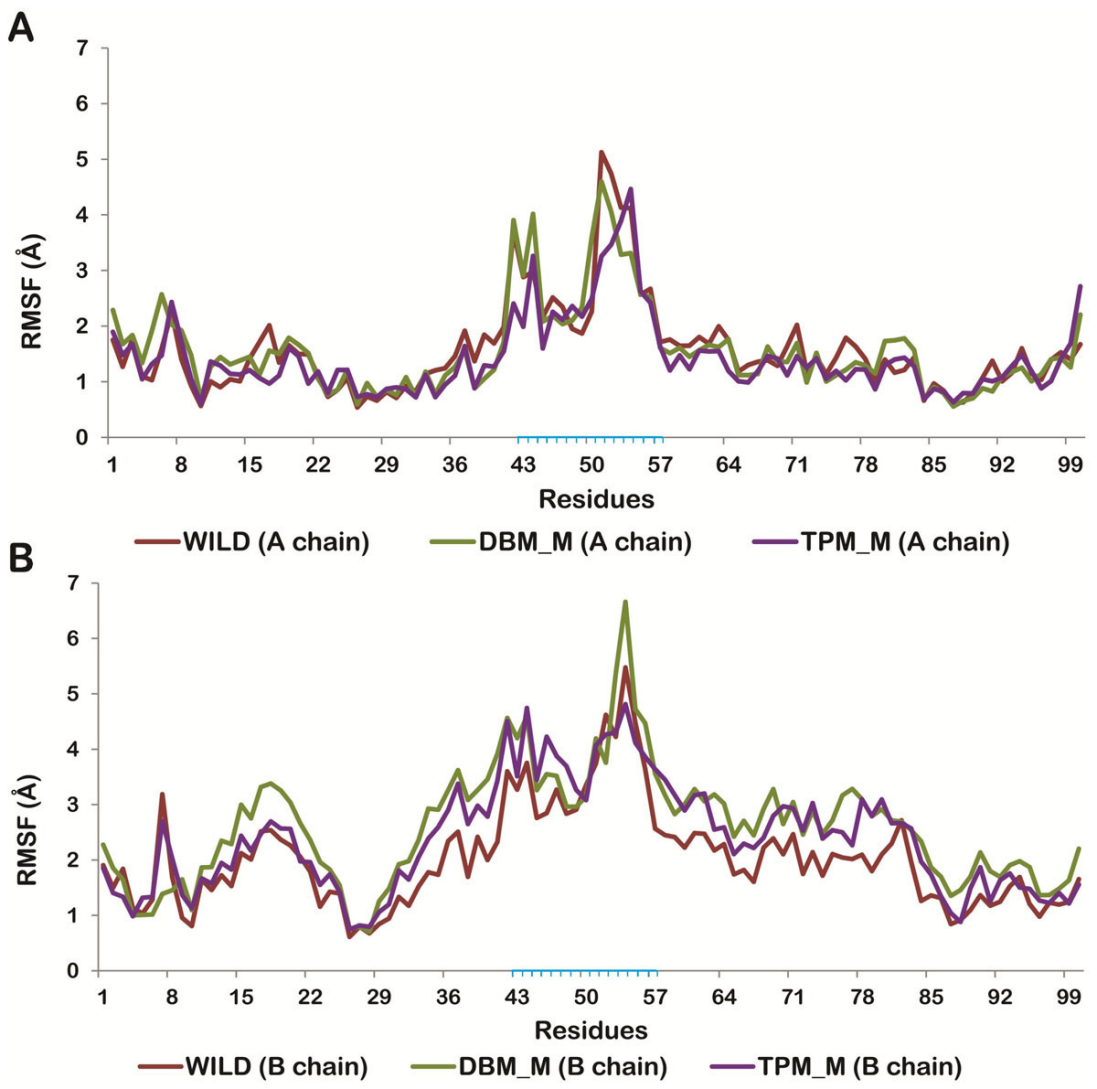
**Residue wise RMS fluctuations of modeled Wild protease (red line), DBM_M (green line) and TPM_M (purple line)**. (A) chain A. (chain B). Residues present in flap region are highlighted in blue.

**Figure 9:**
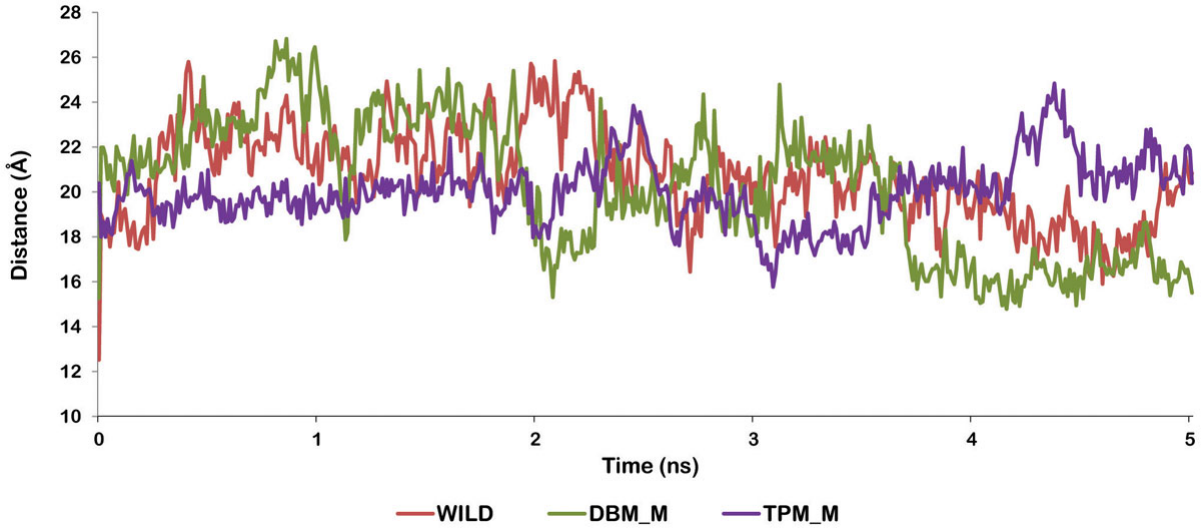
**I50/I50' Distance plot in wild protease (red line), DBM_M (green line) and TPM _M (purple line)**.

**Figure 10:**
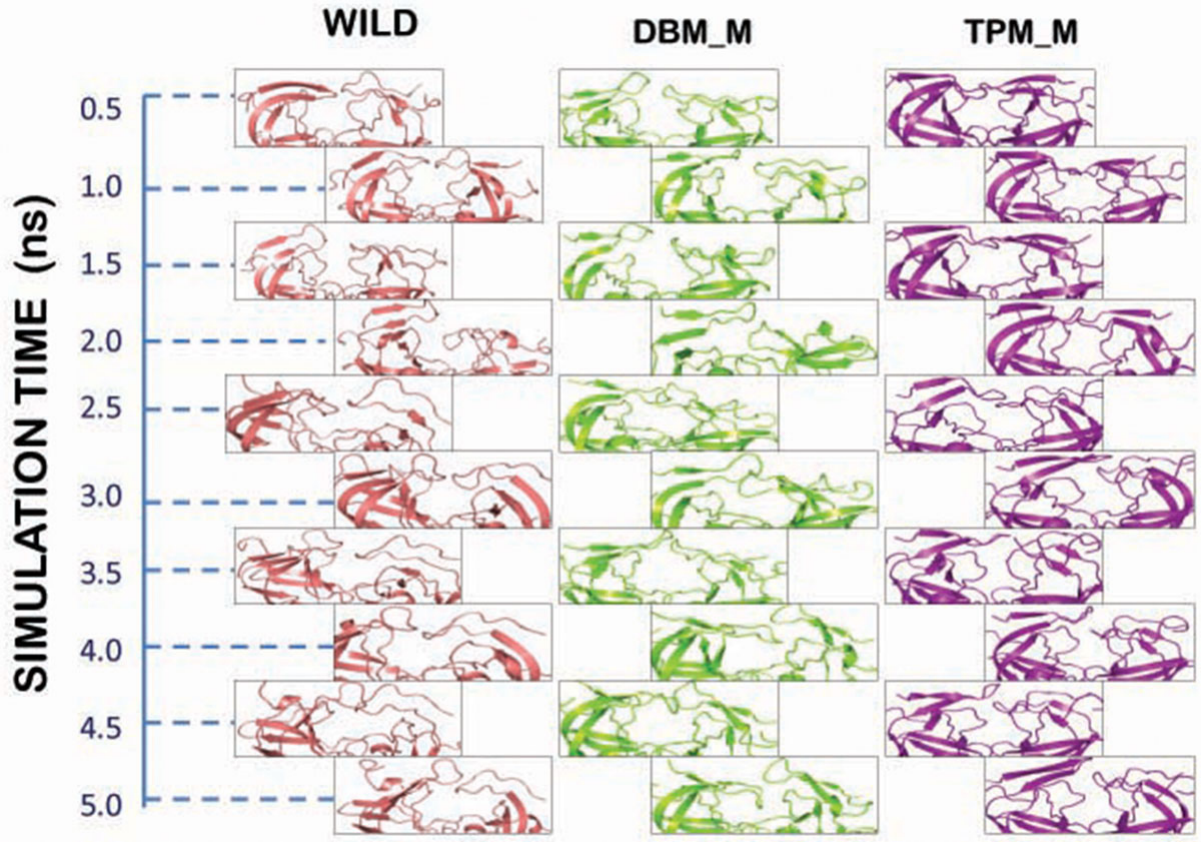
**Flap mutant of Wild protease (1HVP), DBM_M and TPM_M**.

To study the effect of V77I mutation along with L33F and K20T on the flap movements of HIV protease, we considered the semi open modeled structure of HIV-1PR (PDB:1HVP) [33]. The molecular dynamics simulation was performed to view the flap opening mechanism of mutants and compare them with the wild type protease. The structure was processed and mutated as mentioned before. The mutants of this protease structure are abbreviated as DBM_M (V77I, L33F) and TPM_M (V77I, L33F, and K20T). The wild type and mutated structures were stable throughout the simulations trajectory of 5 ns. RMSD of DBM_M was found to be least (0.3) suggesting its more stable nature in comparison to wild type (0.45) and TPM_M (0.53). The RMSD trajectories of all the three structures are shown in Figure 7. The RMSF plots of both the chains were plotted for wild type, DBM_M and TPM_M (Figure 8A, B). Though not much of a difference was observed between the mutants and wild type, the B chain of DBM_M was found to be highly flexible with the RMSF of flap residues reaching as high as 6.66 Å. This indicated wider opening of the flap residues of DBM_M. To verify this proposal we calculated the distance between I50(A)-Cα and I50(B)-Cα atoms [47]. The transition between semi open and open conformations of protease flaps is characterized by interaction between I50 residues located on the tips of the flaps. The flaps of DBM_M separated to the maximum distance of 26.8Å between I50 Cα atoms, before 1 ns of simulation time (Figure 9). This separation occurred early in wild type protease at around 400 ps with the separation of 25.7Å between I50 Cα atoms. DBM_M and wild type protease regained their semi-open open conformations after 1.13 ns. The closing of DBM_M after 2.03 ns is not in sync with the wild type protease. The flaps of TPM_M showed an entirely different trend throughout the simulation time. They retained their closed to semi open state for about 2.25 ns. This distance plot suggested that flaps of DBM_M are more flexible in comparison to NFV-susceptible wild type protease and less clinically prominent TPM_M protease. The flap movements of wild type and mutant proteases were visualized after specific time intervals during the simulations and are depicted in Figure 10.

### Putative selective mechanism of resistant mutations

Along with the selective pressure arising with anti-viral treatment, immunological pressure has also been reported as a sound theory behind the emergence of resistant mutations in HIV-1 PR. This is also credible in case of V77I mutation as it is located as an anchoring residue in the epitope recognized by HLA-A3 [48], (nonamer-LIGPTPVNI). The score representing probability of binding and presenting of the peptide by HLA-A3 was seen to be decreased to a small extent, suggesting that the emergence of mutation V77I is preferentially due to selective pressures imposed by anti-viral therapy, and less likely due to immunological pressure.

## Conclusion

The present study explains the molecular mechanisms through which the V77I mutation in HIV-1 protease cause resistance towards NFV. Since this is a non-active site accessory mutation and clinically occurs with other resistant mutants, we have considered two types of mutant proteases double (DBM) V77I-L33F and triple mutant (TPM) V77I-L33F-K20T in the study. NFV showed a lower binding affinity towards DBM, and this mutant was found to be more stable than the wild type. The flap opening conformation of DBM_M suggested wider separation of flaps and higher flexibility, thus showing that the effect of mutation on the equilibrium of closed and semiopen conformations of protease which could be one reason behind the resistance showed by DBM. Further, the increased cavity size of DBM explained decreased binding affinity of NFV for the mutant protease being accounted by reduced contact surface area. TPM showed increased affinity towards drug and this explains the reason behind its less clinical prevalence. The decreased pocket size and stable flaps suggested that the combination of three mutations made HIV-1 PR non-resistant towards NFV and hence would not have been selected by nature. However the clinical presence of these three mutations together suggests that the mutant protease in nature may have been made resistant due to the presence of other mutations.

## Competing interests

The authors declare that they have no competing interests.

## Authors' contributions

AnG, SJ and AG designed the methods and experimental setup. AnG, SJ and SG carried out the implementation of the various methods and assisted by RJ and DW. AnG, SJ, SG and AG wrote the manuscript. All authors have read and approved the final manuscript.

## Declarations

AG would like to acknowledge financial support from Department of Science and Technology, Government of India towards publication of this article.

This article has been published as part of *BMC Bioinformatics *Volume 16 Supplement 19, 2015: Brazilian Symposium on Bioinformatics 2014. The full contents of the supplement are available online at http://www.biomedcentral.com/bmcbioinformatics/supplements/16/S19
